# Antenatal Psychosocial Resources and Obstetric Context Associated with Clinically Diagnosed Postpartum Depression: A Prospective Cohort Study

**DOI:** 10.3390/healthcare14142156

**Published:** 2026-07-17

**Authors:** Caner Yeşiloğlu, Sinem Çetin Demirtaş, Lut Tamam, Süleyman Cansun Demir, Mehmet Emin Demirkol, Özge Keleş Bayer, Aslı Sena Alagöz, Çağla Boyvadoğlu

**Affiliations:** 1 Department of Psychiatry, Çukurova University Faculty of Medicine, Adana 01330, Türkiye; 2 Department of Obstetrics and Gynecology, Çukurova University Faculty of Medicine, Adana 01330, Türkiye

**Keywords:** postpartum depression, antenatal depression, social support, parity, obstetric factors

## Abstract

**Highlights:**

**What are the main findings?**

- Clinically diagnosed postpartum depression was identified in 29.4% of women at 6 weeks postpartum.
- Lower perceived social support and higher parity remained associated with postpartum depression in an exploratory two-domain adjusted model, while antenatal psychological distress, lower resilience, pain catastrophizing, and obstetric complexity contributed to a broader vulnerability profile.

**What are the implications of the main findings?**

- Antenatal assessment of perceived social support and parity may help identify women who require closer postpartum monitoring.
- The findings support an integrated psychosocial–obstetric approach to postpartum depression risk assessment rather than focusing on delivery mode alone.

**Abstract:**

**Background/Objectives:** Postpartum depression (PPD) is a clinically important perinatal mental health condition affecting maternal functioning and early mother–infant interaction. Prospective studies using clinically diagnosed PPD outcomes while jointly assessing antenatal psychosocial resources and obstetric context remain limited. This study examined antenatal psychosocial and obstetric factors associated with clinically diagnosed PPD at 6 weeks postpartum. **Methods:** This prospective cohort study included 85 pregnant women assessed at ≥24 weeks of gestation in a tertiary university hospital. Women receiving psychiatric treatment during pregnancy or meeting DSM-5 criteria for current depressive disorder at baseline were excluded; women with elevated antenatal depressive symptom scores but no current clinical depressive disorder were retained. Antenatal depressive symptoms, perceived stress, perceived social support, psychological resilience, pain catastrophizing, and clinician–patient relational empathy were assessed using standardized measures. Obstetric and neonatal data were extracted from medical records. PPD was assessed at 6 weeks postpartum using a structured DSM-5-based psychiatric interview. Group comparisons, univariable logistic regression analyses, and an exploratory two-domain adjusted logistic regression model were performed. **Results:** PPD was diagnosed in 25 women (29.4%). Women who developed PPD had higher parity, more frequent maternal obstetric comorbidity, lower infant birth weight and head circumference, higher depressive symptom severity, perceived stress, and pain catastrophizing, and lower perceived social support and psychological resilience. In the exploratory adjusted model, greater perceived social support was associated with lower odds of PPD (adjusted odds ratio [aOR] = 0.58, 95% confidence interval [CI]: 0.35–0.96), whereas higher parity was associated with higher odds (aOR = 2.24, 95% CI: 1.17–4.28). **Conclusions:** Clinically diagnosed PPD was associated with antenatal psychosocial vulnerability and obstetric context. Antenatal assessment of perceived social support, parity, and broader psychosocial vulnerability indicators may help identify women requiring closer postpartum monitoring.

## 1. Introduction

Postpartum depression (PPD) is a common and clinically important mental health condition during the perinatal period. Prevalence estimates vary according to population characteristics, timing of assessment, and diagnostic method, but PPD remains a major contributor to maternal morbidity [1,2]. It has been associated with impaired maternal functioning, difficulties in mother–infant bonding, reduced breastfeeding, and adverse developmental outcomes in children [2,3].

PPD is best understood as a multifactorial condition that develops through interactions among biological vulnerability, antenatal psychological symptoms, psychosocial resources, and perinatal stressors [2,4]. Recent evidence further supports the relevance of antenatal psychosocial vulnerability, including low perceived social support, stress-related symptoms, and reduced resilience, in shaping perinatal mental health risk [5,6,7]. However, prospective studies that use clinically confirmed PPD outcomes and evaluate psychosocial resources together with obstetric context remain limited [2,4,8].

Antenatal depressive symptoms are among the most consistent factors associated with PPD. Prospective evidence indicates that depressive symptoms during pregnancy are associated with later postpartum depressive symptoms, even when antenatal symptoms are identified through screening rather than clinical diagnosis [5,6]. These findings support the clinical relevance of monitoring depressive symptoms during pregnancy, including subthreshold presentations.

Psychosocial resources are also relevant to postpartum mental health. Perceived social support, including partner, family, and informal support, has been associated with lower postpartum depressive symptomatology and may support maternal adjustment by strengthening coping capacity and maternal self-efficacy [7,8]. Recent systematic and longitudinal evidence also indicates that low social support during pregnancy is associated with poorer antenatal mental health and may contribute to vulnerability extending into the postpartum period [9,10].

Pregnancy also involves psychological and somatic stressors that may increase vulnerability to postpartum mood disturbance. Perceived stress, pregnancy-related anxiety, and antenatal depressive symptoms have been associated with adverse maternal and child outcomes and with later postnatal depressive symptoms [4,6,9]. Pain catastrophizing, characterized by rumination, magnification, and helplessness in response to pain, may also be clinically relevant in the perinatal period because catastrophic pain appraisal can intensify the subjective experience of labor pain [10].

The quality of patient–clinician interaction may also be relevant during pregnancy as part of the broader interpersonal context of perinatal care. Relational factors have been discussed in models of PPD risk, and perceived clinician empathy represents a measurable component of healthcare communication [11,12]. However, direct evidence linking antenatal clinician empathy to clinically diagnosed PPD remains limited [13]. Therefore, clinician–patient relational empathy was included in the present study primarily as an exploratory relational healthcare construct rather than as a variable with an established predictive role.

Psychological resilience may further shape postpartum adjustment by supporting adaptive coping and cognitive flexibility during the transition to motherhood. Prospective evidence suggests that lower resilience during pregnancy is associated with greater vulnerability to antepartum and postpartum depressive symptoms, whereas higher resilience may buffer the impact of antenatal psychological risk factors [14,15].

Overall, existing evidence supports an integrated approach to PPD that considers antenatal symptom burden, psychosocial resources, and obstetric context [2,16]. The present study was not designed to examine relapse or persistence of antenatal major depressive disorder. Rather, by excluding women with current clinical depressive disorder during pregnancy while retaining those with elevated or subthreshold depressive symptoms, we aimed to evaluate whether antenatal psychosocial vulnerability markers are associated with clinically diagnosed PPD emerging after childbirth. This approach is clinically relevant because women who do not meet diagnostic criteria for depression during pregnancy may nevertheless show vulnerability through higher depressive symptom burden, perceived stress, lower social support, reduced resilience, pain catastrophizing, or obstetric-contextual factors. 

Accordingly, the present study aimed to examine antenatal psychosocial and obstetric factors associated with clinically diagnosed PPD in a tertiary obstetric cohort. We hypothesized that antenatal depressive symptom severity, perceived stress, pain catastrophizing, lower perceived social support, and lower psychological resilience would be associated with PPD. Given the limited number of PPD events, adjusted analyses were planned as exploratory and parsimonious, with attention to overfitting and multicollinearity.

## 2. Materials and Methods

### 2.1. Study Design

We conducted a prospective cohort study at a tertiary university hospital. Consecutive pregnant women who presented to the perinatology clinic between August and September 2025 and were at 24 weeks of gestation or later were invited to participate. Participants were assessed during pregnancy and followed until 6 weeks postpartum. Antenatal psychological and psychosocial data were collected before delivery, and PPD status was assessed at 6 weeks postpartum using a structured clinical psychiatric interview based on DSM-5 criteria. The psychological and psychosocial scales were administered only during the antenatal assessment and were not re-administered during the postpartum follow-up. Because the antenatal assessment included multiple standardized instruments and required a relatively long evaluation time, repeating the full psychosocial battery during early postpartum follow-up was not considered feasible for many newly delivered mothers. Therefore, the postpartum assessment was limited to the clinical diagnostic evaluation of PPD.

Eligible participants were women aged 18 years or older who had sufficient command of Turkish to complete the study questionnaires and provided written informed consent. Exclusion criteria were cognitive impairment, current psychiatric treatment during pregnancy, and current depressive disorder identified during the baseline antenatal psychiatric assessment. Baseline psychiatric assessment was conducted clinically by a psychiatrist using DSM-5 criteria. Beck Depression Inventory was not used as an exclusion criterion unless the participant met DSM-5 criteria for a current depressive disorder; therefore, women with elevated or subthreshold antenatal depressive symptoms but no current clinical depressive disorder were retained in the cohort. Of 110 women initially enrolled, 25 were excluded: 6 were receiving psychiatric treatment during pregnancy, 7 had incomplete antenatal self-report data, 7 delivered at external institutions, and 5 met criteria for current depressive disorder at baseline. The final analyzed cohort included 85 women who completed both the antenatal assessment and postpartum diagnostic evaluation. No participants in the final analyzed cohort were lost to postpartum follow-up after inclusion in the analyzed sample. 

Seven women delivered at external institutions and therefore did not have complete obstetric and postpartum diagnostic follow-up data available within our hospital records. Because of the small number of these participants, formal statistical comparison with the final analyzed cohort was not considered reliable. Available baseline characteristics were reviewed descriptively and are presented in Supplementary Table S5. 

Obstetric and neonatal data were extracted from medical records after delivery. Obstetric variables included mode of delivery, parity, and maternal obstetric comorbidity documented in obstetric records. Maternal obstetric comorbidity was coded as a binary variable indicating the presence or absence of any documented maternal medical or pregnancy-related maternal obstetric condition. Conditions coded under this variable included maternal obesity, maternal anemia, gestational diabetes, pregestational/type 2 diabetes, hypertensive disorders or elevated blood pressure, hypothyroidism, preeclampsia history, pituitary adenoma/tumor history, hepatitis B carrier status, urinary tract infection, maternal gastric bypass history, Behçet disease, and other documented maternal medical conditions. Fetal indications, delivery indications, previous cesarean delivery, parity/nulliparity, tubal ligation request, labor status, and fetal growth or amniotic fluid abnormalities alone were not coded as maternal obstetric comorbidity. Because condition-specific severity grading was not systematically available in the study records, this variable was analyzed as a binary marker of maternal obstetric comorbidity. Mode of delivery was classified as vaginal delivery or cesarean delivery for analysis. Neonatal variables included infant sex, birth weight, birth length, head circumference, and Apgar score at 5 min.

Study size was considered during the design stage. Based on conventional sample size planning assumptions, a two-sided independent-samples comparison using α = 0.05, 80% power, and a small-to-moderate standardized effect size of d = 0.40 would require approximately 99 participants per group under balanced group allocation. The effect-size assumption was selected to represent a value between Cohen’s conventional small and medium standardized effects, and 80% power was used as a conventional planning threshold [17,18]. This target could not be fully achieved because of prospective clinical follow-up constraints, eligibility restrictions, incomplete antenatal questionnaires, delivery outside the study center, and baseline exclusions. Therefore, the study should be interpreted as an exploratory prospective cohort rather than as a fully powered confirmatory study.

### 2.2. Statistical Analysis

All statistical analyses were performed using IBM SPSS Statistics, version 26.0 (IBM Corp., Armonk, NY, USA). Statistical significance was set at *p* < 0.05 using two-sided tests. PPD was defined as a binary outcome based on a clinical psychiatric interview conducted at 6 weeks postpartum.

The distribution of continuous variables was evaluated using visual inspection of histograms and Q–Q plots together with the Shapiro–Wilk test. Normally distributed variables are presented as mean and standard deviation, whereas non-normally distributed variables are presented as median and interquartile range. Categorical variables are presented as frequencies and percentages.

Group comparisons between women with and without PPD were performed using the independent-samples *t* test for normally distributed continuous variables and the Mann–Whitney U test for non-normally distributed continuous variables. Categorical variables were compared using the chi-square test or Fisher’s exact test, as appropriate. Associations among antenatal psychological and psychosocial measures were examined using Spearman correlation coefficients. Available sociodemographic and clinical variables relevant to residual confounding were compared between women with and without PPD in supplementary exploratory analyses using Mann–Whitney U or Fisher’s exact tests, as appropriate; these analyses were not used for covariate selection in the primary adjusted model. 

Univariable logistic regression analyses were conducted to examine the association between antenatal psychosocial and obstetric variables and PPD. Before logistic regression analyses, continuous psychological scale scores, including the Beck Depression Inventory, Perceived Stress Scale, Multidimensional Scale of Perceived Social Support, Brief Resilience Scale, Pain Catastrophizing Scale, and Consultation and Relational Empathy Measure, were standardized as z scores by subtracting the sample mean and dividing by the sample standard deviation. Therefore, odds ratios for these continuous psychological measures represent the change in odds of PPD per 1-standard deviation increase in the corresponding scale score. Parity was entered as an unstandardized continuous variable, and its odds ratio represents the change in odds of PPD per one-unit increase.

#### Rationale for the Exploratory Adjusted Model

The adjusted analysis was planned as an exploratory two-domain model including one psychosocial resource variable and one obstetric-context variable. This parsimonious approach was used because only 25 women had clinically diagnosed PPD, limiting the number of variables that could be included without increasing the risk of overfitting.

Perceived social support was selected to represent the psychosocial resource domain because low perceived support has been consistently associated with postpartum depressive symptomatology and maternal adjustment [7,8]. It is also clinically interpretable, potentially modifiable, and less directly symptom-based than depressive symptom severity or perceived stress. Psychological resilience was interpreted as an individual resource that overlapped with distress-related vulnerability markers in this small sample, whereas perceived social support was selected as the interpersonal resource domain because it is more directly actionable in antenatal psychosocial support planning. Depressive symptom severity, perceived stress, psychological resilience, and pain catastrophizing were treated as clinically relevant but interrelated markers of antenatal psychosocial vulnerability, based on previous evidence linking these domains with perinatal mental health outcomes [6,9,10,14,15]. Therefore, these variables were interpreted together as components of a broader antenatal vulnerability profile rather than entered simultaneously into the same adjusted model. The observed weak-to-moderate correlations among these measures also supported this cautious approach, indicating partial overlap among related but clinically distinct psychosocial domains.

Parity was selected to represent the obstetric-context domain because it is objectively defined, routinely recorded, and clinically interpretable as a marker of broader family, caregiving, and obstetric context. Previous findings regarding parity and perinatal depression are heterogeneous, with studies supporting both multiparity and first-time motherhood as context-dependent markers of risk. Therefore, parity was interpreted as an obstetric-context marker rather than as a direct causal exposure. Maternal obstetric comorbidity was not included in the primary adjusted model because it was a heterogeneous binary composite variable, and condition-specific severity was not systematically graded.

Maternal age was evaluated in a sensitivity analysis because it may be clinically related to parity. Collinearity diagnostics for the sensitivity model did not indicate problematic multicollinearity. Model calibration was evaluated using the Hosmer–Lemeshow goodness-of-fit test, and discrimination was assessed using the area under the receiver operating characteristic curve [19].

### 2.3. Measures

#### 2.3.1. Sociodemographic and Obstetric Data

Sociodemographic and obstetric characteristics were recorded using a researcher-developed structured data collection form. Sociodemographic variables included maternal age and place of residence. Obstetric variables included parity, mode of delivery, and pregnancy-related maternal obstetric comorbidity. 

#### 2.3.2. Postpartum Depression

PPD was assessed at 6 weeks postpartum using a structured clinical psychiatric interview based on DSM-5 criteria [20]. Interviews were conducted by a psychiatry faculty member experienced in clinical diagnostic assessment. PPD was analyzed as a binary outcome variable.

#### 2.3.3. Beck Depression Inventory 

Antenatal depressive symptom severity was assessed using the Beck Depression Inventory, a 21-item self-report instrument measuring cognitive, affective, and somatic symptoms of depression during the preceding 2 weeks [21]. Items are scored from 0 to 3, yielding a total score ranging from 0 to 63, with higher scores indicating greater depressive symptom severity. The Turkish version has demonstrated acceptable reliability and validity [22]. 

#### 2.3.4. Perceived Stress Scale 

Perceived stress during pregnancy was assessed using the 10-item Perceived Stress Scale. The scale measures the extent to which individuals appraise situations in their lives as stressful, with items scored from 0 to 4 and higher scores indicating greater perceived stress [23]. The Turkish adaptation has demonstrated acceptable reliability and validity [24]. 

#### 2.3.5. Multidimensional Scale of Perceived Social Support 

Perceived social support was assessed using the Multidimensional Scale of Perceived Social Support, a 12-item self-report scale measuring perceived support from family, friends, and a significant other [25]. Items are rated on a 7-point Likert scale, with higher scores indicating greater perceived social support. The Turkish version has demonstrated acceptable factorial validity and reliability [26]. 

#### 2.3.6. Brief Resilience Scale 

Psychological resilience was assessed using the Brief Resilience Scale, a brief self-report instrument developed to measure an individual’s perceived ability to recover from stress and adversity [27]. The scale provides a concise assessment of resilience as the capacity to “bounce back”. Higher scores indicate greater psychological resilience. The Turkish version of the Brief Resilience Scale has demonstrated acceptable reliability and validity [28].

#### 2.3.7. Pain Catastrophizing Scale

Pain-related cognitive appraisal was assessed using the Pain Catastrophizing Scale, a 13-item self-report instrument that measures catastrophic thoughts and feelings related to pain [29]. The scale includes rumination, magnification, and helplessness dimensions, with higher total scores indicating greater pain catastrophizing. The Turkish version has demonstrated acceptable reliability and validity [30]. 

#### 2.3.8. Consultation and Relational Empathy Measure

Perceived relational empathy in healthcare interactions was assessed using the Consultation and Relational Empathy Measure, a 10-item patient-reported instrument evaluating perceived empathy, understanding, and relational communication during clinical encounters [12]. Items are rated on a 5-point scale, with higher scores indicating greater perceived relational empathy. The Turkish version has demonstrated acceptable reliability and validity [31].

## 3. Results

At follow-up, 25 of 85 participants (29.4%) met DSM-5 diagnostic criteria for PPD. Baseline sociodemographic, obstetric, and neonatal characteristics of the cohort, stratified by PPD status, are summarized in Table 1. Women who developed PPD were older and had higher parity compared with those who did not develop PPD. Maternal obstetric comorbidity was also more frequent in the PPD group. Cesarean delivery was numerically more common among women with PPD, but the difference did not reach statistical significance. Infants of women who developed PPD had lower birth weight and head circumference. No statistically significant differences were observed between groups with respect to residence, infant sex, Apgar score at 5 min, or birth length. Maternal obstetric comorbidity was recorded in 18 women (21.2%), including 9 women in the PPD group and 9 women in the non-PPD group. This variable represented a heterogeneous group of maternal medical and pregnancy-related maternal obstetric conditions.

Group comparisons of antenatal psychological and psychosocial scale scores are presented in Table 2. Women who developed PPD had higher antenatal depressive symptom severity, perceived stress, and pain catastrophizing scores than women who did not develop PPD. They also had lower perceived social support and psychological resilience scores. Consultation and Relational Empathy Measure scores were lower in the PPD group numerically, but this difference was not statistically significant.

Correlations among antenatal psychological and psychosocial measures across the total sample are presented in Table 3. Beck Depression Inventory scores were positively correlated with Perceived Stress Scale and Pain Catastrophizing Scale scores and negatively correlated with Multidimensional Scale of Perceived Social Support and Brief Resilience Scale scores. Perceived Stress Scale scores were negatively correlated with Multidimensional Scale of Perceived Social Support and Brief Resilience Scale scores and positively correlated with Pain Catastrophizing Scale scores. Multidimensional Scale of Perceived Social Support scores were positively correlated with Brief Resilience Scale and Consultation and Relational Empathy Measure scores. These correlations were generally weak to moderate in magnitude.

Univariable logistic regression analyses of antenatal psychosocial and obstetric factors associated with PPD are shown in Table 4. Higher perceived stress was associated with higher odds of PPD, whereas higher psychological resilience was associated with lower odds of PPD. Higher Beck Depression Inventory and Pain Catastrophizing Scale scores showed positive associations that did not reach statistical significance in univariable logistic regression. Although perceived social support did not reach conventional statistical significance in univariable logistic regression, it was retained in the exploratory adjusted model according to the two-domain analytic rationale described above. Among obstetric variables, maternal obstetric comorbidity and higher parity were associated with higher odds of PPD, whereas cesarean delivery was not statistically significant.

In exploratory analyses of cesarean delivery subtypes (Table S2), PPD occurred in 62.5% of women who underwent acute cesarean delivery and 32.1% of those who underwent non-acute or pre-planned cesarean delivery. Although the point estimate suggested higher odds of PPD after acute cesarean delivery, the association was not statistically significant. In the present cohort, cesarean delivery was numerically more frequent among women with postpartum depression but was not statistically significant. 

The exploratory two-domain adjusted logistic regression model is presented in Table 5. This exploratory adjusted model should not be interpreted as identifying the only clinically relevant antenatal factors associated with PPD. Rather, perceived social support and parity were selected as two clinically interpretable and minimally overlapping variables representing psychosocial resources and obstetric context. Other antenatal variables, including depressive symptom severity, perceived stress, psychological resilience, and pain catastrophizing, were considered part of a broader psychosocial vulnerability profile. In the adjusted model, greater perceived social support was associated with lower odds of PPD (aOR = 0.58, 95% CI: 0.35–0.96, *p* = 0.033), whereas higher parity was associated with higher odds of PPD (aOR = 2.24, 95% CI: 1.17–4.28, *p* = 0.015). The model had an area under the receiver operating characteristic curve of 0.70, a Nagelkerke R^2^ of 0.14, and a Hosmer–Lemeshow goodness-of-fit test *p* value of 0.186.

A sensitivity analysis was performed by additionally adjusting the exploratory model for maternal age because women who developed PPD were older and had higher parity. Collinearity diagnostics did not indicate problematic multicollinearity among perceived social support, parity, and maternal age; all variance inflation factors were close to 1 and well below conventional thresholds. In this sensitivity analysis, greater perceived social support remained associated with lower odds of PPD (aOR = 0.54, 95% CI: 0.32–0.91, *p* = 0.021), and higher parity remained associated with higher odds of PPD after additional adjustment for maternal age (aOR = 2.64, 95% CI: 1.33–5.24, *p* = 0.005). Maternal age was also associated with PPD in this sensitivity model (aOR = 1.16, 95% CI: 1.04–1.29, *p* = 0.008). The sensitivity analysis is presented in Table S3.

Supplementary exploratory comparisons of available sociodemographic and clinical variables are presented in Table S4. Educational level, employment status, family psychiatric history, smoking status, chronic disease, previous psychiatric history, and income below the minimum wage did not differ significantly between women with and without PPD. All participants were married; therefore, marital status could not be evaluated as a source of between-group variation.

## 4. Discussion

In the present study, clinically diagnosed PPD was identified in 25 of 85 women (29.4%) at 6 weeks postpartum. This prevalence should be interpreted in relation to both the tertiary obstetric setting and the use of a structured clinical diagnostic assessment rather than screening instruments alone. Participants were recruited from a tertiary university hospital, where pregnancy-related complexity and referral-related clinical risk may be higher than in community-based or routine obstetric populations. Therefore, the relatively high prevalence of PPD observed in this study may partly reflect referral-center characteristics and the broader obstetric context of the analyzed sample. Diagnostic interview-based estimates of PPD also vary across populations and may differ from estimates derived from self-report screening scales [32,33]. These setting-related factors should be considered when interpreting the external validity of the findings. Overall, the findings support a multifactorial interpretation of PPD in which antenatal psychological symptoms, psychosocial resources, and obstetric context are considered together [2,16].

The temporal ordering of the assessments is central to the interpretation of these findings. Antenatal depressive symptom severity, perceived stress, perceived social support, psychological resilience, and pain catastrophizing were assessed during pregnancy, whereas PPD was diagnosed after childbirth using a structured clinical psychiatric interview. Therefore, the findings should not be interpreted as indicating that psychosocial difficulties emerged only after delivery. Rather, women who later met diagnostic criteria for PPD had already shown a measurable antenatal psychosocial vulnerability profile before childbirth. From a vulnerability–resource perspective, antenatal depressive symptoms, perceived stress, and pain catastrophizing may be considered vulnerability markers, whereas perceived social support and psychological resilience may represent psychosocial resources that support adaptation during the transition to motherhood. This integrated interpretation is consistent with prospective evidence linking antenatal depressive symptoms to later postnatal depression [6]. Perceived stress during pregnancy has also been discussed as relevant to maternal adjustment and later postnatal depressive symptoms [9]. Conversely, perceived social support has been associated with lower postpartum depressive symptomatology and greater maternal self-efficacy [7,8]. Psychological resilience may further buffer vulnerability to antepartum and postpartum depressive symptoms [14,15]. These domains are also consistent with broader multifactorial models of PPD [16]. Pain catastrophizing may reflect a cognitive–affective appraisal style in response to childbirth-related pain and bodily threat [10]. These findings are compatible with a peripartum vulnerability framework; however, because postpartum psychosocial measures were not repeated, this study cannot determine whether PPD reflected persistence, escalation, or new onset of depressive morbidity.

In the parsimonious adjusted model, lower perceived social support was associated with higher odds of clinically diagnosed PPD. This finding is consistent with previous evidence showing that global and relationship-specific perceived support are associated with lower postpartum depressive symptomatology [7]. Social support may contribute to maternal adjustment by reducing perceived burden, facilitating practical assistance, and strengthening maternal self-efficacy during the early postpartum period [8]. From a behavioral perspective, perceived social support may function as a modifiable interpersonal resource that supports coping and adaptation during the transition to motherhood. Because perceived social support is clinically interpretable and potentially modifiable, its antenatal assessment may help identify women who could benefit from closer postpartum follow-up or additional psychosocial support. However, this finding should be interpreted as an exploratory adjusted association rather than as evidence that perceived social support alone can be used for formal risk prediction. In routine antenatal care, brief assessment of perceived social support, parity-related caregiving demands, and subthreshold psychosocial distress may provide a pragmatic way to identify women who require closer postpartum monitoring. These factors could be incorporated as contextual risk indicators within existing antenatal psychosocial assessment pathways, prompting targeted follow-up, referral to psychosocial support services, or earlier postpartum mental health review when clinically indicated. However, such use should remain supportive and clinical rather than algorithmic until externally validated risk assessment models are available.

Higher parity was also associated with higher odds of clinically diagnosed PPD in the parsimonious adjusted model. This finding should be interpreted cautiously because parity-related findings in the perinatal depression literature are heterogeneous. A recent systematic review and meta-analysis identified multiparity as a factor associated with perinatal depression, whereas a large multinational postpartum study reported higher postpartum depressive symptom burden among first-time mothers [34,35]. Therefore, in the present study, parity should be viewed as a context-dependent marker rather than as a direct causal or biological exposure. From a behavioral and psychosocial perspective, higher parity may reflect greater caregiving load, childcare responsibilities, cumulative role strain, previous obstetric or birth-related experiences, and changes in the availability or perceived adequacy of practical and emotional support. These factors may increase the subjective burden of adaptation during pregnancy and the early postpartum period, particularly when perceived social support is low. This interpretation is consistent with multifactorial models of PPD and with evidence linking subjective birth experience, relational factors, and delivery-related context with postpartum mental health [11,16].

Women who developed PPD were also older and had higher parity than women who did not develop PPD. Because maternal age and parity are clinically related but not interchangeable obstetric-context variables, the parity finding was further examined in a sensitivity analysis additionally adjusted for maternal age. Higher parity remained associated with PPD in this sensitivity analysis, suggesting that the observed association was not explained by maternal age alone. However, this should not be interpreted as definitive evidence that parity is an independent predictor. Maternal age was also associated with PPD in the sensitivity model, and age, parity, obstetric history, family structure, caregiving demands, and unmeasured psychosocial stressors may be closely linked in clinical practice. This finding suggests that maternal age may be an informative covariate in this cohort, although the small sample size and exploratory design preclude firm conclusions regarding its independent contribution. Obstetric evidence also indicates that the implications of maternal age may differ according to parity and that age–parity combinations may be associated with different pregnancy and neonatal outcomes [36,37]. Thus, a cautious interpretation is that parity may function as a clinically available marker of broader obstetric, family, and caregiving context rather than as a simple causal factor.

Antenatal depressive symptom severity, perceived stress, psychological resilience, and pain catastrophizing should be interpreted as related but distinct components of an antenatal psychosocial vulnerability profile rather than as isolated explanatory variables. Women who later developed clinically diagnosed PPD had higher depressive symptom severity during pregnancy, and previous prospective evidence has linked antenatal depressive symptoms with later postnatal depression [6]. This finding is clinically relevant because women with current DSM-5 depressive disorder at baseline were excluded, whereas women with elevated or subthreshold depressive symptom scores who did not meet criteria for current depressive disorder were retained. Thus, the present findings suggest that clinically meaningful vulnerability may be detectable during pregnancy even below the threshold of current depressive disorder; however, this interpretation should remain cautious because the study did not include repeated postpartum symptom measures.

Perceived stress was also higher among women who developed PPD. This finding is consistent with literature indicating that stress, anxiety, and depressive symptoms during pregnancy are relevant to maternal adjustment and later postnatal depressive symptoms [9]. In the present study, perceived stress should therefore be viewed as part of the broader antenatal vulnerability profile rather than as an independent factor established by the adjusted model. Psychological resilience showed the opposite pattern: women who developed PPD had lower resilience during pregnancy. This is consistent with prospective studies suggesting that resilience during pregnancy may buffer vulnerability to antepartum and postpartum depressive symptoms [14,15]. More recent systematic evidence also supports the relevance of resilience as a psychosocial resource associated with better perinatal mental health [38]. However, because resilience overlapped conceptually and statistically with depressive symptom severity and other psychosocial measures, it was not retained in the parsimonious adjusted model. Therefore, lower resilience should be interpreted as a clinically relevant component of antenatal psychosocial vulnerability, not as an independent predictor established by this study.

Pain catastrophizing was higher among women who developed PPD and may represent a cognitive–affective dimension of antenatal vulnerability. The Pain Catastrophizing Scale captures rumination, magnification, and helplessness in response to pain, and obstetric evidence has linked higher pain catastrophizing with greater labor pain and analgesia-related outcomes [10]. Additional perinatal evidence suggests that pain-related appraisal and childbirth-related distress may be relevant to postpartum psychological adjustment [39]. In the present study, pain catastrophizing was not retained in the adjusted model because of the limited number of PPD events and overlap among psychosocial measures. Accordingly, depressive symptom severity, perceived stress, lower resilience, and pain catastrophizing should be interpreted together as exploratory indicators of antenatal psychosocial vulnerability. Their exclusion from the adjusted model does not indicate clinical irrelevance; rather, it reflects the need to avoid overfitting and to preserve the exploratory two-domain modeling strategy. Larger prospective studies with repeated antenatal and postpartum psychosocial assessments are needed to clarify the independent and combined contributions of these domains.

Clinician–patient relational empathy was numerically lower among women who developed PPD, but this difference did not reach statistical significance. This finding should not be interpreted as evidence that healthcare communication is irrelevant to postpartum mental health. Relational factors have been included in broader models of PPD risk, and clinician empathy represents a measurable component of healthcare communication [11,12,13]. However, the Consultation and Relational Empathy Measure was assessed at a single antenatal time point in the present study. Therefore, it may not fully reflect continuity of care, repeated clinical encounters, disclosure of emotional distress, or access to timely psychosocial support during pregnancy and after childbirth. Future prospective studies should examine healthcare communication as a longitudinal relational process rather than as a single-point patient-reported exposure.

Maternal obstetric comorbidity was associated with PPD in univariable analysis, but this finding should be interpreted cautiously. This variable represented a heterogeneous binary marker of maternal medical and pregnancy-related obstetric conditions, and condition-specific severity grading was not systematically available. Therefore, the observed association may reflect broader maternal obstetric complexity rather than the effect of any single condition. This interpretation is consistent with multifactorial models of PPD, in which obstetric complications may be interpreted alongside antenatal psychological vulnerability, perceived stress, social support, and subjective childbirth experience [2,16]. In contrast, cesarean delivery was numerically more frequent among women with PPD but did not reach statistical significance. Previous evidence regarding cesarean delivery and PPD has been mixed, and this relationship appears to depend on contextual factors such as emergency or acute cesarean delivery, perceived control during childbirth, obstetric complications, and subjective birth experience rather than delivery mode alone [11,16,40]. Accordingly, the present findings do not support interpreting cesarean delivery itself as an independent risk factor for PPD. Larger prospective studies with more detailed assessment of obstetric indications, maternal comorbidities, perceived control, and subjective birth experience are needed to clarify the role of delivery-related context.

In group comparisons, infants of women who developed PPD had lower birth weight and head circumference, whereas Apgar score at 5 min, infant sex, and birth length did not differ significantly between groups. These neonatal findings should be interpreted cautiously because neonatal variables were not included in the parsimonious adjusted model and may reflect broader obstetric complexity rather than independent associations with PPD. This finding should not be interpreted as evidence that neonatal factors are unrelated to maternal mental health. Rather, the neonatal variables available in this study were limited to basic physical and clinical indicators, whereas previous studies suggest that subjective childbirth experience, preterm birth, neonatal intensive care unit-related stress, and infant sleep problems may be more directly relevant to postpartum psychological distress [40,41,42]. Given the temporal and clinical complexity of neonatal outcomes, these variables were not modeled as mediators or confounders in the present exploratory analysis; instead, they were treated as descriptive neonatal indicators that may reflect obstetric complexity and should be interpreted cautiously in light of multiple group comparisons and the limited number of PPD events.

Several limitations should be considered when interpreting the findings. First, the sample size was modest, particularly because only 25 women were diagnosed with PPD. Although study size was considered during the design stage, the planned sample target could not be achieved because of prospective clinical follow-up constraints, eligibility restrictions, incomplete antenatal questionnaires, delivery outside the study center, and baseline exclusions. Therefore, the study may have been underpowered to detect small-to-moderate associations, and non-significant findings should not be interpreted as evidence that clinically meaningful associations are absent. The limited number of PPD events also restricted the complexity of multivariable modeling. For this reason, the adjusted model was intentionally specified as a parsimonious and exploratory two-domain model rather than as a full multivariable prediction model. The adjusted effect estimates should therefore be interpreted cautiously, particularly where confidence intervals were relatively wide. The limited number of events also precluded reliable testing of interaction terms, such as potential interactions between perceived social support and parity.

Second, residual and unmeasured confounding remain possible. Supplementary exploratory comparisons of available sociodemographic and clinical variables, including educational level, employment status, family psychiatric history, smoking status, chronic disease, previous psychiatric history, and income below the minimum wage, did not differ significantly between women with and without PPD. However, these analyses cannot replace a fully adjusted multivariable model. Several potentially relevant factors were not systematically available, including marital or partner relationship quality, pregnancy planning, prior PPD, intimate partner violence, breastfeeding, sleep disruption, NICU-related stress, birth trauma, and subjective childbirth experience. These factors may influence both perceived social support and vulnerability to PPD. Therefore, the adjusted associations should be interpreted as exploratory and potentially influenced by residual confounding.

Third, most antenatal psychological and psychosocial variables were assessed using self-report instruments. Although validated Turkish versions of the scales were used, self-administered questionnaires may be influenced by current emotional state, subjective appraisal, recall-related bias, and shared measurement-method effects. Reporting bias and common-method variance may therefore have contributed to the observed associations among depressive symptom severity, perceived stress, perceived social support, psychological resilience, and pain catastrophizing. This limitation is partly balanced by the fact that PPD itself was assessed using a structured DSM-5-based clinical psychiatric interview rather than a self-report screening scale alone.

Fourth, selection and attrition bias may limit external validity. The exclusion of women receiving psychiatric treatment during pregnancy, women with current depressive disorder at baseline, participants with incomplete questionnaires, and women who delivered outside the study center may have resulted in a more clinically stable and adherent analyzed sample than would be expected in routine obstetric practice. Conversely, recruitment from a tertiary university hospital may have increased the representation of women with greater obstetric complexity. Available baseline characteristics of women who delivered externally were summarized descriptively, but the small number of these participants precluded reliable formal comparison. Therefore, selection may have operated in more than one direction, and the observed prevalence and adjusted associations should be generalized cautiously, particularly to women receiving antenatal care in community-based or lower-level healthcare settings.

Fifth, psychological and psychosocial variables were not reassessed after childbirth. Depressive symptom severity, perceived stress, perceived social support, psychological resilience, pain catastrophizing, and clinician–patient relational empathy were measured during pregnancy only. Therefore, the study identifies antenatal factors associated with later clinically diagnosed PPD but does not directly evaluate within-person changes in psychosocial functioning from pregnancy to the postpartum period. In addition, PPD was assessed at a single early postpartum time point at 6 weeks. Although this timing allowed standardized clinical diagnostic assessment during the early postpartum period, later-onset PPD may have been missed. Finally, the single-center design and observational nature of the study preclude causal inference.

These limitations should be balanced against several strengths. The prospective design ensured that antenatal psychosocial variables were assessed before the postpartum diagnostic evaluation. PPD was defined using a structured DSM-5-based clinical psychiatric interview rather than a self-report screening scale alone, strengthening the clinical validity of the primary outcome assessment. Follow-up was complete within the analyzed sample, and obstetric and neonatal data were extracted from medical records. The study also evaluated psychosocial resources, cognitive–affective vulnerability markers, and obstetric context together, which supports a broader behavioral interpretation of antenatal vulnerability. Overall, the findings suggest that lower perceived social support, higher parity, antenatal psychological distress, lower resilience, pain-related cognitive vulnerability, and pregnancy-related obstetric complexity may represent clinically relevant signals for closer postpartum monitoring. However, these findings should be considered exploratory and should not be interpreted as a validated prediction model or as sufficient evidence for a formal antenatal risk-prediction or monitoring strategy without confirmation in larger, more diverse, externally validated prospective studies.

## 5. Conclusions

In this prospective study, clinically diagnosed PPD was associated with antenatal psychosocial vulnerability and obstetric context. Lower perceived social support and higher parity remained associated with PPD in the exploratory two-domain adjusted model; however, these estimates should be interpreted as exploratory adjusted associations rather than as definitive independent predictors or as a validated prediction model. The findings also suggest that antenatal depressive symptom severity, perceived stress, lower psychological resilience, and pain catastrophizing may represent clinically relevant components of a broader antenatal psychosocial vulnerability profile, even when current DSM-5 depressive disorder is not present at baseline.

Perceived social support may represent a clinically interpretable and potentially modifiable psychosocial resource that can be assessed during antenatal care. In contrast, parity should be viewed as a marker of a broader obstetric, family, and caregiving context rather than as a simple causal factor. Taken together, assessment of perceived social support, parity, and broader psychosocial vulnerability indicators may help identify women who could benefit from closer postpartum monitoring or additional psychosocial support. These findings should be considered hypothesis-generating and should not be interpreted as sufficient evidence for formal antenatal risk prediction.

Further longitudinal studies with larger and more diverse samples, multicenter recruitment, longer postpartum follow-up, external validation, and repeated psychosocial assessments during pregnancy and after childbirth are needed to clarify trajectories of antenatal vulnerability, determine the independent and combined contributions of psychosocial and obstetric-context factors, and evaluate whether these domains can inform evidence-based antenatal monitoring approaches.

## Figures and Tables

**Table 1:** Sociodemographic, obstetric, and neonatal characteristics of the participants.

Variable	Total (N = 85)	No PPD (n = 60)	PPD (n = 25)	*p* Value	Effect Size
Age, years	29.8 ± 5.3	28.9 ± 4.8	31.9 ± 5.9	0.015	d = 0.59
Residence, n (%)				0.344	V = 0.12
- Village/Town	45 (52.9%)	34 (56.7%)	11 (44.0%)		
- City	40 (47.1%)	26 (43.3%)	14 (56.0%)		
Parity	1 (1–2)	1 (1–2)	2 (1–2)	0.038	r_rb = 0.27
Maternal obstetric comorbidity, n (%)	18 (21.2%)	9 (15.0%)	9 (36.0%)	0.042	V = 0.23
Mode of delivery, n (%)				0.148	V = 0.18
- Vaginal	49 (57.6%)	38 (63.3%)	11 (44.0%)		
- Cesarean	36 (42.4%)	22 (36.7%)	14 (56.0%)		
Apgar score at 5 min	9 (8–9)	9 (8–9)	9 (8–9)	0.309	r_rb = −0.12
Infant sex, female, n (%)	41 (48.2%)	31 (51.7%)	10 (40.0%)	0.352	V = 0.11
Birth weight, g	3109 ± 417	3171 ± 379	2962 ± 474	0.034	d = −0.51
Birth length, cm	48 (48–50)	48 (48–50)	48 (47–49)	0.517	r_rb = −0.09
Head circumference, cm	34 (33–35)	34 (33–35)	33 (33–34)	0.026	r_rb = −0.30

Note: Values are presented as mean ± SD, median [IQR], or n (%) as appropriate. PPD, postpartum depression. Effect sizes are presented to indicate the magnitude of between-group differences. Cohen’s d was used for normally distributed continuous variables, rank-biserial correlation for non-normally distributed continuous variables, and Cramer’s V for categorical variables.

**Table 2:** Comparison of antenatal psychological scale scores by postpartum depression status.

Scale	No PPD (n = 60)	PPD (n = 25)	*p* Value	Effect Size
Perceived Stress Scale	19.07 ± 9.29	24.32 ± 9.53	0.021	d = 0.56
Multidimensional Scale of Perceived Social Support	72 (64–78)	62 (52–71)	0.016	r_rb = −0.33
Brief Resilience Scale	19.93 ± 5.06	16.08 ± 5.21	0.002	d = −0.76
Pain Catastrophizing Scale	13 (5–24)	23 (16–36)	0.047	r_rb = 0.28
Consultation and Relational Empathy Measure	47 (42–50)	43 (37–50)	0.116	r_rb = −0.21
Beck Depression Inventory	8 (5–12)	14 (9–20)	0.002	r_rb = 0.43

Note: Values are presented as mean ± SD or median [IQR] as appropriate. Effect sizes are presented to indicate the magnitude of between-group differences. Cohen’s d was used for normally distributed continuous variables, and rank-biserial correlation was used for non-normally distributed continuous variables.

**Table 3:** Correlations among antenatal psychological and psychosocial measures.

Scale	MSPSS	BRS	PCS	CARE	BDI
Perceived Stress Scale	−0.30 *	−0.42 **	0.39 **	−0.16	0.53 **
Multidimensional Scale of Perceived Social Support	-	0.37 **	−0.28 *	0.28 *	−0.36 **
Brief Resilience Scale		-	−0.34 *	0.12	−0.55 **
Pain Catastrophizing Scale			-	−0.19	0.33 *
Consultation and Relational Empathy Measure				-	−0.14

Note: Spearman correlation coefficients are shown. * *p* < 0.05; ** *p* < 0.01. Abbreviations: BDI, Beck Depression Inventory; BRS, Brief Resilience Scale; CARE, Consultation and Relational Empathy Measure; MSPSS, Multidimensional Scale of Perceived Social Support; PCS, Pain Catastrophizing Scale; PPD, postpartum depression; PSS, Perceived Stress Scale.

**Table 4:** Univariable logistic regression analyses of antenatal psychosocial and obstetric factors associated with postpartum depression.

Variable	OR	95% CI	*p* Value
Beck Depression Inventory	1.51	0.96–2.38	0.077
Multidimensional Scale of Perceived Social Support	0.70	0.44–1.10	0.119
Brief Resilience Scale	0.45	0.26–0.78	0.004
Pain Catastrophizing Scale	1.58	0.99–2.53	0.057
Consultation and Relational Empathy Measure	0.79	0.50–1.25	0.315
Perceived Stress Scale	1.78	1.08–2.95	0.025
Cesarean delivery	2.20	0.85–5.67	0.104
Maternal obstetric comorbidity	3.19	1.08–9.40	0.036
Parity	1.85	1.01–3.37	0.046

Note: OR, odds ratio; CI, confidence interval. Continuous psychological scale scores were standardized as z scores before logistic regression; therefore, ORs for these measures are reported per 1-standard deviation increase. The OR for parity is reported per one-unit increase. For categorical variables, the reference categories were vaginal delivery for cesarean delivery and absence of maternal obstetric comorbidity for maternal obstetric comorbidity.

**Table 5:** Exploratory two-domain adjusted logistic regression model of postpartum depression.

Variable	Adjusted OR	95% CI	*p* Value
Multidimensional Scale of Perceived Social Support	0.58	0.35–0.96	0.033
Parity	2.24	1.17–4.28	0.015

Note: aOR, adjusted odds ratio; CI, confidence interval. The aOR for the Multidimensional Scale of Perceived Social Support is reported per 1-standard deviation increase because the scale score was standardized as a z score before logistic regression. The aOR for parity is reported per one-unit increase. The model was specified as an exploratory two-domain adjusted model because only 25 women were diagnosed with postpartum depression; therefore, estimates should be interpreted as exploratory adjusted associations rather than as definitive independent predictors or as a validated prediction model.

## Data Availability

The data are not publicly available due to privacy and ethical restrictions but may be made available from the corresponding author upon reasonable request, subject to institutional approval.

## Supplementary Materials

The following supporting information can be downloaded at: https://www.mdpi.com/article/10.3390/healthcare14142156/s1, Table S1: Indications for cesarean delivery; Table S2: Acute vs. non-acute cesarean delivery and postpartum depression; Table S3: Sensitivity analysis additionally adjusted for maternal age; Table S4: Sociodemographic and clinical variables by postpartum depression status; Table S5: Sociodemographic and clinical variables of women included in the final cohort and women who delivered at external institutions.

## Abbreviations

aOR	Adjusted odds ratio
AUC	Area under the receiver operating characteristic curve
BDI	Beck Depression Inventory
BRS	Brief Resilience Scale
CARE	Consultation and Relational Empathy Measure
CI	Confidence interval
DSM-5	Diagnostic and Statistical Manual of Mental Disorders, Fifth Edition
IQR	Interquartile range
MSPSS	Multidimensional Scale of Perceived Social Support
NICU	Neonatal intensive care unit
OR	Odds ratio
PCS	Pain Catastrophizing Scale
PPD	Postpartum depression
PSS	Perceived Stress Scale
SD	Standard deviation

## References

[1] Gavin N.I., Gaynes B.N., Lohr K.N., Meltzer-Brody S., Gartlehner G., Swinson T. (2005). Perinatal depression: A systematic review of prevalence and incidence. Obstet. Gynecol..

[2] O’Hara M.W., McCabe J.E. (2013). Postpartum depression: Current status and future directions. Annu. Rev. Clin. Psychol..

[3] Stewart D.E., Vigod S. (2016). Postpartum depression. N. Engl. J. Med..

[4] Howard L.M., Molyneaux E., Dennis C.L., Rochat T., Stein A., Milgrom J. (2014). Non-psychotic mental disorders in the perinatal period. Lancet.

[5] Tanuma-Takahashi A., Tanemoto T., Nagata C., Yokomizo R., Konishi A., Takehara K., Ishikawa T., Yanaihara N., Samura O., Okamoto A. (2022). Antenatal screening timeline and cutoff scores of the Edinburgh Postnatal Depression Scale for predicting postpartum depressive symptoms in healthy women: A prospective cohort study. BMC Pregnancy Childbirth.

[6] Milgrom J., Gemmill A.W., Bilszta J.L., Hayes B., Barnett B., Brooks J., Ericksen J., Ellwood D., Buist A. (2008). Antenatal risk factors for postnatal depression: A large prospective study. J. Affect. Disord..

[7] Dennis C.L., Letourneau N. (2007). Global and relationship-specific perceptions of support and the development of postpartum depressive symptomatology. Soc. Psychiatry Psychiatr. Epidemiol..

[8] Leahy-Warren P., McCarthy G., Corcoran P. (2012). First-time mothers: Social support, maternal parental self-efficacy and postnatal depression. J. Clin. Nurs..

[9] Dunkel Schetter C., Tanner L. (2012). Anxiety, depression and stress in pregnancy: Implications for mothers, children, research, and practice. Curr. Opin. Psychiatry.

[10] Peralta F.M., Condon L.P., Torrez D., Neumann K.E., Pollet A.L., McCarthy R.J. (2024). Association of pain catastrophizing with labor pain and analgesia consumption in obstetrical patients. Int. J. Obstet. Anesth..

[11] Smorti M., Ponti L., Pancetti F. (2019). A comprehensive analysis of postpartum depression risk factors: The role of socio-demographic, individual, relational, and delivery characteristics. Front. Public Health.

[12] Mercer S.W., Maxwell M., Heaney D., Watt G.C.M. (2004). The consultation and relational empathy (CARE) measure: Development and preliminary validation and reliability of an empathy-based consultation process measure. Fam. Pract..

[13] Derksen F., Bensing J., Lagro-Janssen A. (2013). Effectiveness of empathy in general practice: A systematic review. Br. J. Gen. Pract..

[14] Tobe H., Kita S., Hayashi M., Umeshita K., Kamibeppu K. (2020). Mediating effect of resilience during pregnancy on the association between maternal trait anger and postnatal depression. Compr. Psychiatry.

[15] Hain S., Oddo-Sommerfeld S., Bahlmann F., Louwen F., Schermelleh-Engel K. (2016). Risk and protective factors for antepartum and postpartum depression: A prospective study. J. Psychosom. Obstet. Gynaecol..

[16] Yim I.S., Tanner Stapleton L.R., Guardino C.M., Hahn-Holbrook J., Dunkel Schetter C. (2015). Biological and psychosocial predictors of postpartum depression: Systematic review and call for integration. Annu. Rev. Clin. Psychol..

[17] Cohen J. (1992). A power primer. Psychol. Bull..

[18] Lakens D. (2022). Sample size justification. Collabra Psychol..

[19] Hosmer D.W., Lemeshow S., Sturdivant R.X. (2013). Applied Logistic Regression.

[20] American Psychiatric Association (2013). Diagnostic and Statistical Manual of Mental Disorders.

[21] Beck A.T., Ward C.H., Mendelson M., Mock J., Erbaugh J. (1961). An inventory for measuring depression. Arch. Gen. Psychiatry.

[22] Hisli N. (1989). Beck Depresyon Envanterinin üniversite öğrencileri için geçerliği, güvenirliği. Psikol. Derg..

[23] Cohen S., Kamarck T., Mermelstein R. (1983). A global measure of perceived stress. J. Health Soc. Behav..

[24] Eskin M., Harlak H., Demirkıran F., Dereboy Ç. (2013). The adaptation of the Perceived Stress Scale into Turkish: A reliability and validity analysis. New Symp. J..

[25] Dahlem N.W., Zimet G.D., Walker R.R. (1991). The Multidimensional Scale of Perceived Social Support: A confirmation study. J. Clin. Psychol..

[26] Eker D., Arkar H., Yaldız H. (2001). Çok Boyutlu Algılanan Sosyal Destek Ölçeği’nin gözden geçirilmiş formunun faktör yapısı, geçerlik ve güvenirliği. Türk Psikiyatr. Derg..

[27] Smith B.W., Dalen J., Wiggins K., Tooley E., Christopher P., Bernard J. (2008). The Brief Resilience Scale: Assessing the ability to bounce back. Int. J. Behav. Med..

[28] Doğan T. (2015). Kısa Psikolojik Sağlamlık Ölçeği’nin Türkçe uyarlaması: Geçerlik ve güvenirlik çalışması. J. Happiness Well-Being.

[29] Sullivan M.J.L., Bishop S.R., Pivik J. (1995). The Pain Catastrophizing Scale: Development and validation. Psychol. Assess..

[30] Ugurlu M., Karakas Ugurlu G., Erten S., Caykoylu A. (2017). Validity of Turkish form of Pain Catastrophizing Scale and modeling of the relationship between pain-related disability with pain intensity, cognitive, and emotional factors. Psychiatry Clin. Psychopharmacol..

[31] Erzurumlu M., Ozcelik H., Akdeniz M., Kavukcu E., Avci H.H. (2025). Adaptation of the Consultation and Relational Empathy Measure to Turkish. Behav. Sci..

[32] Bai Y., Li Q., Cheng K.K., Caine E.D., Tong Y., Wu X., Gong W. (2023). Prevalence of postpartum depression based on diagnostic interviews: A systematic review and meta-analysis. Depress. Anxiety.

[33] Lyubenova A., Neupane D., Levis B., Wu Y., Sun Y., He C., Krishnan A., Bhandari P.M., Negeri Z., Imran M. (2021). Depression prevalence based on the Edinburgh Postnatal Depression Scale compared to Structured Clinical Interview for DSM Disorders classification: Systematic review and individual participant data meta-analysis. Int. J. Methods Psychiatr. Res..

[34] Bradshaw H., Riddle J.N., Salimgaraev R., Zhaunova L., Payne J.L. (2022). Risk factors associated with postpartum depressive symptoms: A multinational study. J. Affect. Disord..

[35] Yang K., Wu J., Chen X. (2022). Risk factors of perinatal depression in women: A systematic review and meta-analysis. BMC Psychiatry.

[36] Wang Y., Tanbo T., Åbyholm T., Henriksen T. (2011). The impact of advanced maternal age and parity on obstetric and perinatal outcomes in singleton gestations. Arch. Gynecol. Obstet..

[37] Dai J., Shi Y., Wu Y., Guo L., Lu D., Chen Y., Wang Y., Lai H., Kong X. (2023). The interaction between age and parity on adverse pregnancy and neonatal outcomes. Front. Med..

[38] Hajure M., Alemu S.S., Abdu Z., Tesfaye G.M., Workneh Y.A., Dule A., Hussen M.A., Wedajo L.F., Gezimu W. (2024). Resilience and mental health among perinatal women: A systematic review. Front. Psychiatry.

[39] Zeng Y., Tan C.W., Sultana R., Chua T.-E., Chen H.Y., Sia A.T.H., Sng B.L. (2020). Association of pain catastrophizing with postnatal depressive states in nulliparous parturients: A prospective study. Neuropsychiatr. Dis. Treat..

[40] Coo S., García M.I., Mira A. (2023). Examining the association between subjective childbirth experience and maternal mental health at six months postpartum. J. Reprod. Infant. Psychol..

[41] Ionio C., Colombo C., Brazzoduro V., Mascheroni E., Confalonieri E., Castoldi F., Lista G. (2016). Mothers and fathers in NICU: The impact of preterm birth on parental distress. Eur. J. Psychol..

[42] Hiscock H., Wake M. (2001). Infant sleep problems and postnatal depression: A community-based study. Pediatrics.

