# Supplementary material for: Antenatal Psychosocial Resources and Obstetric Context Associated with Clinically Diagnosed Postpartum Depression: A Prospective Cohort Study
Source: Healthcare (Basel). 2026 Jul 17;14(14):2156. doi: 10.3390/healthcare14142156 (PMC13410205; doi:10.3390/healthcare14142156)
Supplement: Supplementary file 1 [file healthcare-14-02156-s001.zip › healthcare-4409100-supplementary.pdf]

Table S1. Indications for Cesarean Delivery

**Indication category**	**n**	**%**
Previous cesarean section	17	47.2
Acute obstetric indication	8	22.2
Maternal medical comorbidity	6	16.7
Fetal anomaly/screening risk	3	8.3
Elective maternal request	2	5.6
Total	36	100.0

Table S2. Acute vs Non-Acute Cesarean Delivery and Postpartum Depression

**Variable**	**No PPD**	**PPD**	**Total**	**% with PPD**
Acute cesarean delivery	3	5	8	62.5
Non-acute cesarean delivery	19	9	28	32.1

Statistical test: Fisher exact test p=.217; OR for acute versus non-acute cesarean delivery = 3.52, 95% CI 0.68–18.07.

Table S3. Sensitivity Analysis Additionally Adjusted For Maternal Age

**Variable**	**aOR**	**95% CI**	**p value**
Multidimensional Scale of Perceived Social Support	0.54	0.32–0.91	.021
Parity	2.64	1.33–5.24	.005
Maternal Age	1.16	1.04–1.29	.008

Note: aOR, adjusted odds ratio; CI, confidence interval. The Multidimensional Scale of Perceived Social Support score was standardized as a z score before logistic regression; therefore, its aOR is reported per 1-standard deviation increase. Parity and maternal age were entered as unstandardized continuous variables; therefore, their aORs are reported per one-unit increase and per 1-year increase, respectively. This model was performed as a sensitivity analysis only and was not intended to replace the primary parsimonious adjusted model.

Table S4. Sociodemographic and clinical variables by postpartum depression status

**Variables**	**No PPD (n=60)**	**PPD (n=25)**	**p values**
Educational level, median [IQR]	4 [3–6]	5 [4–6]	.071
Married, n (%)	60 (100.0)	25 (100.0)	—
Employed, n (%)	25 (41.7)	9 (36.0)	.808
Family psychiatric history, n (%)	10 (16.7)	1 (4.0)	.163
Smoking, n (%)	7 (11.7)	4 (16.0)	.724
Chronic disease, n (%)	8 (13.3)	8 (32.0)	.066
Previous psychiatric history, n (%)	16 (26.7)	5 (20.0)	.591
Income below minimum wage, n (%)	10 (16.7)	3 (12)	.747

Table S5. Sociodemographic and clinical variables of women included in the final cohort and women who delivered at external institutions

**Variable**	**Final analyzed cohort (n=85)**	**External delivery (n=7)**
Maternal age, years, mean ± SD	29.8 ± 5.3	29.3 ± 5.1
Educational level, median [IQR]	5 [4–6]	6 [5–6]
Number of children, median [IQR]	1 [0–2]	1 [0–2]
Married, n (%)	85 (100.0)	7 (100.0)
Employed, n (%)	34 (40.0)	4 (57.1)
Family psychiatric history, n (%)	11 (12.9)	0 (0.0)
Smoking, n (%)	11 (12.9)	1 (14.3)
Chronic disease, n (%)	16 (18.8)	3 (42.9)
Previous psychiatric history, n (%)	21 (24.7)	2 (28.6)

Note: Values are presented as mean ± SD, median [IQR], or n (%), as appropriate. These comparisons are descriptive only. No formal statistical testing was performed because only seven women delivered at external institutions. External delivery resulted in unavailable complete obstetric and postpartum diagnostic follow-up data within the study institution. PPD, postpartum depression; SD, standard deviation; IQR, interquartile range.
